# 1882. Contact Investigation of Congenital Tuberculosis in a Neonatal Intensive Care Unit in South Korea

**DOI:** 10.1093/ofid/ofad500.1710

**Published:** 2023-11-27

**Authors:** Sojeong Lee, Songhee Namgung, Eunbyeol Jo, Mina Yun, Ja Young Kim, Yumi Lee, Jihye Jeong, Sun Kyung Kim, So-Yeon Park, Young-Ju Lim, Eun Ok Kim, Jiwon Jung, Jina Lee, Byong Sop Lee, Sung-Han Kim

**Affiliations:** Asan Medical Center, Seoul, Seoul-t'ukpyolsi, Republic of Korea; Asan Medical Center, Seoul, Seoul-t'ukpyolsi, Republic of Korea; Asan Medical Center, Seoul, Seoul-t'ukpyolsi, Republic of Korea; Asan Medical Center, Seoul, Seoul-t'ukpyolsi, Republic of Korea; Asan Medical Center, Seoul, Seoul-t'ukpyolsi, Republic of Korea; Asan Medical Center, Seoul, Seoul-t'ukpyolsi, Republic of Korea; Asan Medical Center, Seoul, Seoul-t'ukpyolsi, Republic of Korea; Asan Medical Center, Seoul, Seoul-t'ukpyolsi, Republic of Korea; Asan Medical Center, Seoul, Seoul-t'ukpyolsi, Republic of Korea; Asan Medical Center, Seoul, Seoul-t'ukpyolsi, Republic of Korea; Asan medical center, Songpa-gu, Seoul-t'ukpyolsi, Republic of Korea; Asan Medical Center, Seoul, Seoul-t'ukpyolsi, Republic of Korea; Asan Medical Center, Seoul, Seoul-t'ukpyolsi, Republic of Korea; Asan Medical Center, Seoul, Seoul-t'ukpyolsi, Republic of Korea; Asan medical center, Songpa-gu, Seoul-t'ukpyolsi, Republic of Korea

## Abstract

**Background:**

Congenital tuberculosis (TB) is uncommon and difficult to detect in neonates due to its nonspecific symptoms. We conducted a contact investigation of infants and healthcare workers (HCWs) exposed to a neonate with congenital TB in a neonatal ICU (NICU) in Korea.

**Methods:**

A premature infant born was admitted to NICU on September 16, 2022. On October 24, the infant's mother was diagnosed with miliary TB, and infant’s sputum AFB stain showed 4 positive results. All NICU infants and HCWs during the same period were screened for active pulmonary TB using chest radiography (CXR) immediately. Exposed infants were evaluated with a TST and CXR three months after exposure. Interferon-Gamma Release Assay (IGRA) testing was performed on those with a positive TST or abnormal CXR finding. Prophylactic rifampin was provided to the exposed infants for 3 months, as index had isoniazid-resistant *M. tuberculosis* infection. Exposed HCWs underwent IGRA testing immediately after exposure (1^st^ IGRA) and at 8 to 10 weeks post-exposure (2^nd^ IGRA), and CXR was performed 6 months after exposure.

**Results:**

Five out of 82 exposed infants had positive TST (≥ 10 mm) results, while all 31 infants who underwent IGRA testing had negative results. All five with positive TST had received BCG vaccination a median 105 days before. Of the 119 exposed HCWs, three had a conversion; two had negative results (on annual IGRA testing performed according to the national TB prevention Act) before exposure and positive at 1^st^ IGRA test, and one had negative 1^st^ IGRA test then positive 2^nd^ IGRA test. None had active TB during 6-month follow-up.

**Figure:**
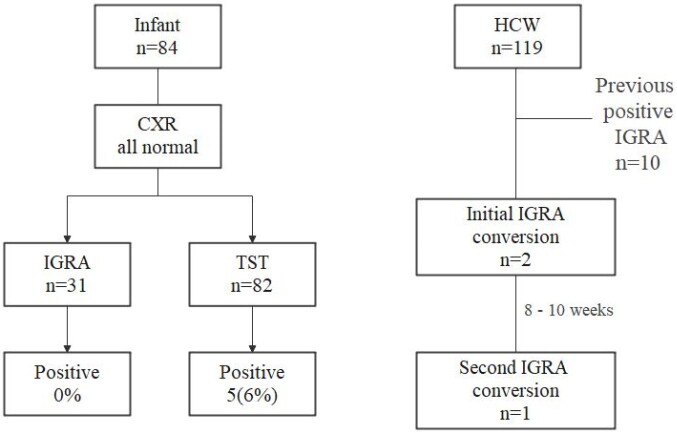

**Conclusion:**

We found that 6% of exposed infants had positive TST results and 0% had positive IGRA, while 2.5% of exposed HCWs had conversion. Considering the possibility of false positive TST results due to prior BCG vaccination, the chance of transmission to the infants would be 0%; otherwise, it would be 6.1%.

**Disclosures:**

**All Authors**: No reported disclosures

